# Overexpression of sulfatase-1 in murine hepatocarcinoma Hca-F cell line downregulates mesothelin and leads to reduction in lymphatic metastasis, both *in vitro* and *in vivo*

**DOI:** 10.18632/oncotarget.11933

**Published:** 2016-09-10

**Authors:** Salma Mahmoud, Mohammed Ibrahim, Ahmed Hago, Yuhong Huang, Yuanyi Wei, Jun Zhang, Qingqing Zhang, Yu Xiao, Jingwen Wang, Munkaila Adam, Yu Guo, Li Wang, Shuting Zhou, Boyi Xin, Wei Xuan, Jianwu Tang

**Affiliations:** ^1^ Department of Pathology, Dalian Medical University, Dalian 116044, China; ^2^ Department of Pathology, School of Medicine and Health Sciences, University for Development Studies, Tamale, Ghana

**Keywords:** sulfatase-1, mesothelin, hepatocellular carcinoma, migration and invasion, lymph node metastasis

## Abstract

Lymphatic vessels function as transport channels for tumor cells to metastasize from the primary site into the lymph nodes. In this experiment we evaluated the effect of Sulfatase-1 (Sulf-1) on metastasis by upregulating it in murine hepatocarcinoma cell line Hca-F with high lymph node metastatic rate of >75%. The study in vitro showed that upregulation of Sulf-1 in Hca-F cells significantly reduced cell proliferation, migration and invasion (p<0.05). Also, the forced expression of Sulf-1 downregulated Mesothelin (Msln) at both the protein and mRNA levels. The experiment in vivo further showed that up-regulation of Sulf-1 with the attendant downregulation of mesothelin delayed tumor growth and decreased lymph node metastasis. In conclusion, our findings show that Sulf-1 is an important tumor suppressor gene in hepatocellular carcinoma (HCC), and its overexpression downregulates Msln and results in a decrease in HCC cell proliferation, migration, invasion, and lymphatic metastasis. This functional relationship between Sulf-1 and Msln could be exploited for the development of a novel liver cancer therapy.

## INTRODUCTION

Hepatocellular carcinoma (HCC) is one of the cancers topping the list of malignancies that are of major global health concern because of its high incidence and case fatality rate. In most cases HCC is diagnosed at an advanced stage, which limits the application of curative treatment [1].

Metastasis is the most important process in cancer progression and it accounts for about 90% of cancer-associated mortalities. To date, the pathogenesis and the genes associated with the cancer metastasis remain poorly understood [2]. The mechanisms by which the malignant tumor cells leave their primary site and invade the lymphatics and proceed to the regional lymph nodes are very complicated and interrelated. The mechanism of cancer cell metastasis has been reported to involve Epithelial Mesenchymal Transition (EMT) and Mesenchymal to Epithelial Transition (MET) [3, 4]. In this process carcinoma cells acquire mesenchymal cell properties (EMT) which will enable them to start the local invasion process followed by metastasis [5]. As soon as the cells reach the target organs, they automatically change their structure to that of an epithelial cell, and this process is known as Mesenchymal to Epithelial Transition (MET) [3, 4] and forms a metastatic lesion in that organ.

In the past few years, our laboratory conducted a study on mouse Hca-F and Hca-P cell lines in order to screen out lymphatic metastasis associated genes. Hca-F cells originated from mouse hepatocellular carcinoma which has been shown to have high lymphatic metastatic rate of >75% [6, 7] compared to Hca-P cells which have <25% metastatic rate. Suppressive subtractive hybridization and gene chip assays were done in these two cell lines and among the genes which were highly expressed in Hca-F cells is mesothelin (Msln). Interestingly, sulfatase-1 (Sulf-1), which is a known tumor suppresser gene in various cancers including hepatocellular carcinoma has been found among the genes that have low expression in the Hca-F cell line [8–11].

Sulfatase-1 (Sulf-1) is a heparin degrading endosulfatase which is known to inhibit tumorigenesis by desulfating cell surface proteoglycans (HSGAGs) and prevents the binding of several ligands including growth factors, interleukins and morphogens such as VEGF, FGF, HGF, EGF and Wnt [12]. Sulf-1 expression inhibited by miR-21 in hepatocellular carcinoma led to increased cell proliferation, migration and invasion. Also inhibition of Sulf-1 in hepatocellular carcinoma facilitated the formation of Epithelial Mesenchymal Transition (EMT) and this increased the chance of distance metastasis by activating AKT/ERK pathways [13]. In human gastric cancer, Sulf-1 was reported to inhibit hedgehog signaling involved in Epithelial Mesenchymal transition (EMT) and at the same time it down regulated metastasis associated genes such as GLI1, PTCH1/2, HHIP, c-myc, CCND1, FOXM1 and bcl2 [14]. In a highly metastatic hepatocellular carcinoma cell line SMMC-7721, Sulf-1 was reported to have inhibited complex formation between hepatocyte growth factor ligand and tyrosine kinase receptor c-met [15] and this further inhibited the activation of ERK, PI3K/AKT pathway.

Mesothelin (Msln) is a 40kDa cell membrane glycoprotein and is known to act as a tumor promoter gene in various malignancies [16]. A study reported that, membrane expression of Msln was associated with lymph node metastasis, blood vessels invasion and increased chance of recurrence in gastric cancer and breast cancer [17, 18]. In breast cancer, Msln was reported to increase the production of matrix metallopeptidase 9 (MMP-9) which is an important factor in blood vessels formation and invasion [19]. Msln expression was reported to be induced by Wnt1 [20] and the stimulation of Wnt1 depends on the 6-O-sulfation status of HSPGs (syndecan-1). Syndecan-1 acts as a core-receptor by increasing the binding affinity of Wnt1 and allows it to stimulate the down ward signaling leading to the promotion of tumor growth and metastasis [12]. Sulf-1 is known to desulfate 6-O- sulfate group from the HSPGs and this leads to a decrease in the binding affinity of Wnt1 to HSPGs. The reduction in the binding affinity of Wnt1 consequently inhibits all the Wnt1 signaling pathways controlling the stimulation of Msln. Therefore, there is a possible link between Sulf-1 and Msln in the reduction of lymphatic metastasis of HCC.

Our results show for the first time that, upregulation of Sulf-1 in Hca-F cells affects the expression of Msln both at the protein and mRNA levels. We have also demonstrated that, upregulation of Sulf-1 in Hca-F cells reduced cell proliferation, migration, invasion and lymph nodes metastasis of hepatocellular carcinoma, both in vitro and in vivo. These results suggest that the ability of Sulf-1 to downregulate Msln may provide a potential therapeutic target in hepatocellular carcinoma.

## RESULTS

### Western blot and qRT-PCR results for the expression of Sulf-1 and Msln

**Figure 1:**
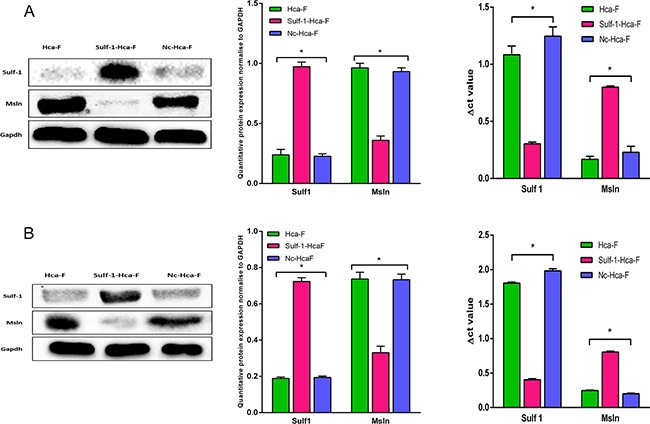
Western blot and qRT-PCR analysis for Sulf-1 and Msln in vitro and in vivo **A.** Western blot analysis for Sulf-1 and Msln relative expression in vitro at the protein and mRNA levels. **B.** Western blot analysis for Sulf-1 and Msln relative expression in in vivo at the protein and mRNA. Statistical significance was observed in Sulf-1-Hca-F group both in protein and mRNA levels. Data are presented in columns as mean ± standard deviation (SD) and the results were statistically significant at *P< 0.05.

Western blot was performed in order to check the expression level of both Sulf-1 and Msln in Hca-F, Sulf-1-Hca-F, and Nc-Hca-F cells. As shown in Figure 1, the expression of Msln in Hca-F and Nc-Hca-F cells both in vitro and in vivo was approximately the same, and the expression of Sulf-1 also did not have significant change between the two controls. However, and quite interestingly, the expression of Sulf-1 following the upregulation increased by 50% while Msln expression decreased concurrently by 50% compared to the two controls.

The levels of mRNA of both Sulf-1 and Msln were also measured in vitro and in vivo by qRT-PCR and normalized to Gapdh, and Δct was used to calculate the values. In the Sulf-1-Hca-F cells the result showed that the Δct values for Sulf-1 in vitro and in vivo were 0.32 and 0.4 while the Δct value for Msln was 0.6 and 0.8, respectively, (*P<0.05). On the other hand, the Δct values for sulf-1 were 1.2 and 1.6 in the in vitro samples and 1.8 and 2.0 in the in vivo samples for Hca-F and Nc-Hca-F cells, respectively. Also the Δct values for Msln were 0.15 and 0.2 in vitro and 0.25 and 0.2 in vivo for Hca-F and Nc-Hca-F cells respectively. This indicate that the mRNA expression level of Sulf-1 in Sulf-1-Hca-F group was higher with a concurrently lower expression of Msln mRNA compared to the two controls, confirming that Sulf-1 upregulation led to Msln downregulation both at the Protein and mRNA levels, Figure 1.

### Upregulation of Sulf-1 decreased cell proliferation ability

**Figure 2:**
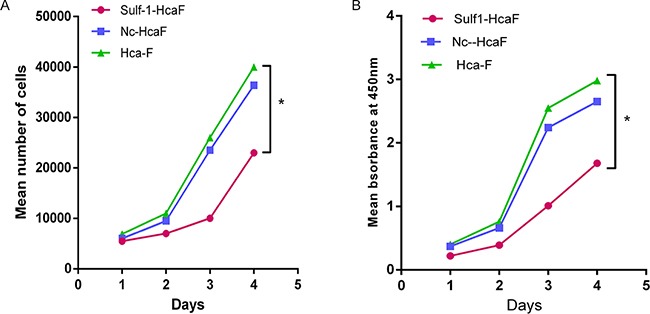
Cell proliferation assay results **A.** cell proliferation measurement using manual cell count and **B.** CCK 8 assay. The curve for Sulf-1-Hca-F cells is seen lagging behind both in the manual cell count and in the CCK8 analysis. This indicates that there was a significant reduction in cell proliferation potential in Sulf-1-Hca-F cells (*P<0.05).

CCK-8 cell proliferation assay was used to evaluate the role of Sulf-1 on proliferation of Hca-F, Sulf-1-Hca-F and Nc-Hca-F cells. The results showed that, there was a significant inhibitory effect on cell proliferation by 60% (*P< 0.05) in the Sulf-1-Hca-F cells compared to the Hca-F and Nc-Hca-F cells as shown in Figure 2. This indicated that Sulf-1 played a tumor suppressor role in the hepatocarcinoma cells.

### Action of Sulf-1 upregulation on cell cycle

**Figure 3:**
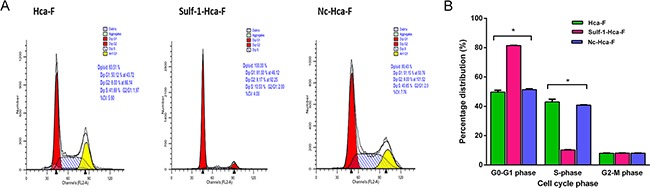
Cell cycle analysis **A.** Cell cycle analysis of Hca-F cells; Sulf-1- Hca-F cells; and Nc- Hca-F cells. Cells were synchronized by growth in 100% confluence with reduced serum for three days after which it was cultured in complete medium for 24hr and were then harvested, washed with PBS, stained with PI and analyzed by flow cytometry. The values represent the number of cells in each phase of the cell cycle as a percentage of the total cells. **B.** Cluster bar chart of the cell cycle showing percentage distributions of the cell cycle phases in the three cell groups. The values were statistically significant at *P< 0.05.

The cell cycle analysis was carried out by PI staining followed by flow cytometry. The results showed that in the Sulf-1-Hca-F cells there was 31% increase in cells accumulation in G0-G1 phase with a concomitant reduction by 32% in the number of cells in S-phase compared to the two controls (*P< 0.05) as shown in Table 1 and Figure 3. This results indicate that upregulation of Sulf-1 inhibits hepatocellular carcinoma cell division.

**Table 1:** Cell cycle percentage distribution

Group	The percentage of cell cycle phase (%)		
	G0-G1	S	G2-M
Hca-F	50.11 ± 0.67	42.88 ± 1.4	8.00 ± 0.85
Sulf-1-Hca-F	81.30 ± 0.07*	10.53 ± 0.03*	8.17 ± 0.02*
Nc-Hca-F	49.15 ± 1.08	41.85 ± 1.09	8.00 ± 0.01

The result for cell cycle analysis in Hca-F, Sulf-1-Hca-F and Nc-Hca-F cells were statistical significant at *P < 0.05.

### Upregulation of Sulf-1 inhibited cell migration and invasion

**Figure 4:**
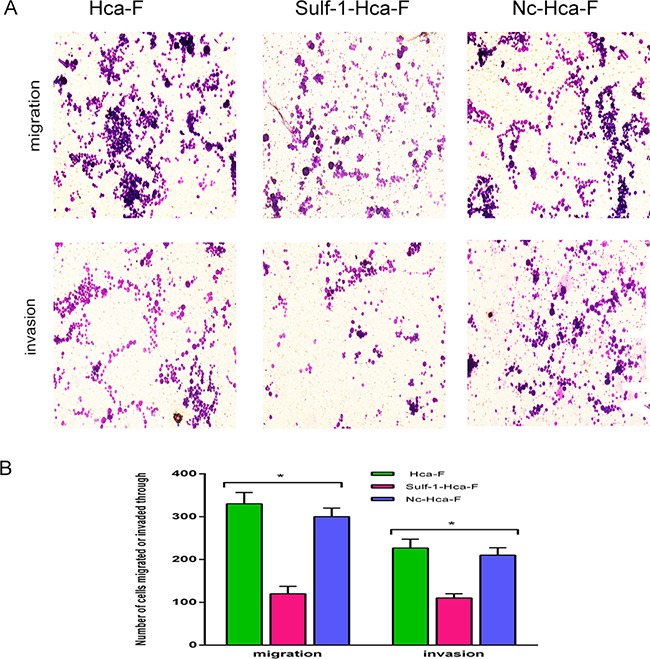
Migration and invasion assay **A.** Transwell migration and invasion assays were performed in Hca-F, Sulf-1-Hca-F and Nc-Hca-F cells. The results show a decrease in both migration and invasion in Sulf-1-Hca-F cells. **B.** Average number of migrating and invading cells presented as a bar chart with mean ± standard deviation (SD). The results were statistically significant at *P< 0.05.

We performed transwell assay to examine the mobility and invasion ability of the stably up regulated Sulf-1-Hca-F and the two controls (Hca-F and Nc-Hca-F cell lines). The Sulf-1-Hca-F cells were shown to have approximately 61% reduction in mobility and 55% reduction in invasion ability. No significant differences were observed in migration and invasion abilities of the Hca-F and Nc-Hca-F groups as shown in Figure 4, (*P> 0.05).

### In vivo analysis

**Figure 5:**
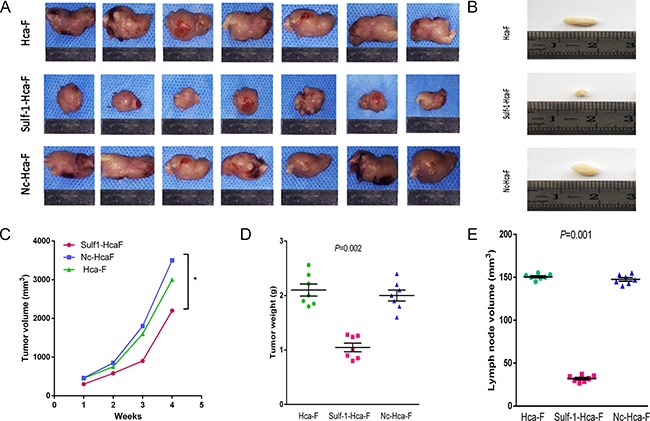
Sizes of tumor and lymph nodes measurement The 615 Chinese inbreed male mice humanely sacrificed and the tumors, **A.** and the lymph nodes, **B.** were dissected. The tumor diameter was measured weekly and the tumor volume, **C.** was calculated with the formula [(length (mm) × width (mm)^2^]/2. The tumors from the Sulf-1-Hca-F group were smaller compared to the two controls. **D.** is tumor weight and E is lymph node volume. The tumors and lymph nodes from Sulf-1-Hca-F group had lower volumes compared to the two controls. The results were statistically significant at *P < 0.05.

After inoculation of the mice with the tumor cells we observed that the group inoculated with the Sulf-1-Hca-F cells had a slower rate of tumor growth and developed smaller tumors compared to the groups that were inoculated with the Hca-F and Nc-Hca-F, Figure 5. Even though all the mice formed tumors following the tumor transplant the Sulf-1-Hca-f group was active compared to the ill-looking cancer burdened control mice in Hca-F and Nc-Hca-F groups.

Also, we observed reduced lymphatic metastatic rate in the Sulf-1-Hca-F compared to the Hca-F and the Nc-Hca-F as shown in Table 2. In Sulf-1-Hca-F group the percentage metastasis of lymph node was low (7%(1/14)) compared to the other two groups where the incidence of lymph node metastasis was 86% (12/14) in Hca-F group and 92% (13/14) in Nc-Hca-F group (*P*<0.05). This result showed that, upregulation of Sulf-1 reduced lymph node metastasis in Hca-F cell lines.

**Table 2:** The role of Sulf-1 upregulation on lymph node metastatic rate

Group	LN	Lymph node metastatic rate level		
		LNM rate	% LNM rate	*P* value
Hca-F	Inguinal	7/7	86	*0.05
	Axillary	5/7		
Sulf-1-Hca-F	Inguinal	1/7	7	
	Axillary	0/7		
Nc-Hca-F	Inguinal	7/7	92	
	Axillary	6/7		

Note: LN refers to lymph node, LNM refers to lymph node metastasis. The difference of lymph node metastatic rate between Hca-F, Sulf-1-Hca-F and Nc-Hca-F groups were statistically significant at *P < 0.05.

### Immunohistochemistry analysis and H&E staining

**Figure 6:**
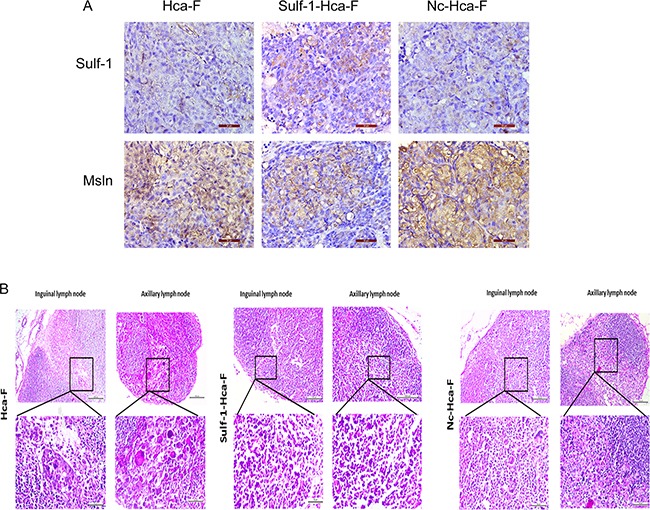
Immunohistochemistry and H&E analysis **A.** Immunohistochemistry Analysis and **B.**, H&E staining in the three different groups: Hca-F, Sulf-1-Hca-F, and Nc-Hca-F cell groups. The Sulf-1 expression is higher in Sulf-1-Hca-F group compared to the Hca-F and Nc-Hca-F groups, and at the same time Msln expression was reduced in Sulf-1-Hca-F group but very high in the controls. The H & E staining showed the lymph nodes from Hca-F and Nc-Hca-F groups had tumor giant cells and abnormal mitotic figures and were generally of high grade compared to that of the Sulf-1-Hca-F group.

The results showed that the intensity of Sulf-1 expression is higher in Sulf-1-Hca-F group compared to the two control groups. At the same time the expression of Msln is reduced in a Sulf-1-Hca-F group compared to the two controls where the expression of Msln was very high. This results showed that forced expression of Sulf-1 led to reduction in the expression of Msln. Dhoot *et al* reported that, Sulf-1 is localized on the cell surface [22] while Msln is expressed on the cell membrane and in the cytoplasm [17]. As shown in Figure 6 above, examination of the lymph nodes with the H&E stain revealed that the lymph nodes from the control groups had tumor giant cells and abnormal mitotic figures, and were generally of high grade compared to that of the sulf1-Hca-F.

## DISCUSSION

Primary tumors undergo some adaptation as a precondition before they can metastasize and be able to survive in the newly invaded environment. This adaptation includes neovascularization that facilitates oxygen as well as nutrient transportation, and the formation of lymphatic vessels which will facilitate the tumor cells' migration from the primary site into the lymph nodes [23]. Factors that increase the differentiation of lymphatics endothelial cells are known to play vital roles in the formation of lymphatic vasculature. These factors include VEGF, HGF, FGF, EDGF, PDGF and IGF. However, the activity of Heparin Sulfate Proteoglycans (HSPGs), a proteoglycan which is known to regulate various genes associated with tumor metastasis, is controlled by Sulf-1 [24]. Sulf-1 is a cell surface endosulfatase which has a unique function of removing 6-O-heparin sulfate groups from HSPGs and alters the binding site for many signaling molecules. The desulfation action of Sulf-1 is known to inhibit many signaling pathways involved in tumor cell differentiation, proliferation, migration, invasion and lymphatics metastasis. It was also shown to increase cancer cells sensitivity to chemotherapeutic drugs [25]. Many published studies reported that Sulf-1 is down regulated in various cancers such as ovarian cancer, breast cancer, head and neck squamous cell carcinoma and renal cell cancer. However, only few have reported the role of Sulf-1 in liver cancer metastasis, and not many of them attempted to directly address LNM of hepatocellular carcinoma [13]. In this current study we have investigated the role of Sulf-1 in tumorigenesis and the role it plays in LNM of hepatocellular cancer. Our data has shown that sulf-1 suppresses hepatocellular cancer and reduces the LNM rate of the tumor.

To further understand the mechanism of Sulf-1 suppression of hepatocellular carcinoma lymphatic metastasis, we investigated it alongside with Msln *gene,* a cell surface glycoprotein which is known to be overexpressed in several cancers including hepatocellular carcinoma. We, also, have previously found Msln to be over expressed in this highly metastatic Hca-F cell line that we have used in this study [10, 11, 9]. This relationship between sulf-1 and Msln has never been explored before by any study. Our data, for the first time, showed that forced expression of Sulf-1 down-regulated Msln expression both at the protein and the mRNA levels. Msln is one of the genes reported to be associated with tumor metastasis. Its expression in pancreatic cancer significantly increased cell proliferation by 90% and cell migration by 300% in vitro [26]. Also overexpression of Msln stimulates the production of IL-6 and this triggers the production of transcriptional protein 3 (stat 3) which results in higher expression of cyclinE/cyclin-dependent kinase (CDK2) complex that accelerates G1-S transition [27]. Furthermore, Msln stimulated PI3/Akt and MAPK/ERK signaling and inhibits pro-apoptotic factors such as Bad and Bax and causes the tumor cells to survive for longer periods, and at the same time promotes the expression of anti-apoptotic gene such as Bcl-2 and Mcl-1 [28, 29]. It was reported that expression of Msln led to an increase lymph node metastasis, blood vessels invasion and lymphatic invasion [30, 31].

**Figure 7:**
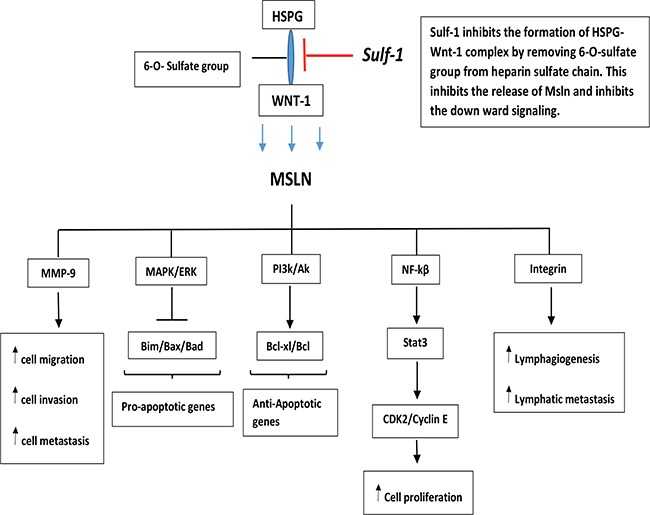
A schematic diagram showing the role of Sulfatase-1 in Carcinogenesis Desulfation action of sulfatase-1 on heparin sulfate proteoglycan (HSPG) leads to the inhibition of formation of HSPG-Wnt1 complex. This action will inhibits the secretion of Mesothelin which is induced by Wnt1stimulation. Reduction of Msln secretion leads to inhibition of the signaling pathway involved in cell proliferation, migration, invasion, lymphatics metastasis, and hence promote cell survival.

The expression of Msln was reported to be controlled by Wnt1/beta-catenin and in order for Wnt 1/beta-catenin to have high binding affinity to Msln, it needed to be attached to syndecan 1, which is an HSPGs with high sulfation status [12, 32]. Another study reported that, overexpression of Msln in breast cancer increased tumor cells lymph node infiltration, and decreased the overall survival rate of the cells by activating ERK1/2-MMP-9 signaling. The ERK1/2-MMP-9 signaling was found to be important in facilitating tumor cell invasion, migration and metastasis [33]. Knockdown of Msln was also reported to silence Wnt/beta-catenin signaling which played important role in Epithelial-Mesenchymal Transition (EMT), an important step in tumor metastasis process [34]. A study reported that luminal membrane expression of Msln both in gastric cancer and colorectal cancer correlated with the lymph node metastasis, blood vessels invasion, lymphatic invasion, recurrence and poor patient outcome [17, 31]. Our data showed that up-regulated Sulf-1 decreased Msln expression, and this could be attributable to the inhibitory effects of Sulf-1 on HSPGs that prevents the downstream intracellular signaling by Wnt, Figure 7.

Tumor Cell over proliferation is one of the important prognostic factors in tumor evolution, and this happens when there is a gain of function mutation in the genes controlling cell proliferation or loss of function mutation in tumor suppressors. Many studies reported that sulfation of HSPGs helps to maintain the stability, and increased the affinity of ligand-receptor binding of Wnt and Hedgehog growth factors [35–37]. Consistently, Sulf-1 is reported to suppress the growth and cell proliferation of gastric cancer cell line MKN28 by inhibiting Hedgehog signaling [14] that results from the removal of the 6-O-sulfate group from HSPGs. Our results showed reduction in cell proliferation in the Sulf-1 up-regulated cells compared to the controls (Figure 2), confirming the tumor suppressor role of Sulf-1 as reported in other studies.

We further observed that there was a significant increase in the accumulation of cells in G0-G1 phase by 31% with an attendant 32% decrease in the S-phase cell number in the Sulf-1 up-regulated group compared to the controls. The in vivo study also showed reduced tumor growth rate in the mice inoculated with Sulf-1-Hac-F. This finding is in agreement with Xu G, *et al.,* who reported that Sulf-1 causes cell cycle arrest in hepatocellular carcinoma by reducing the binding affinity of bFGF to its receptor through HSPGs desulfation. [38].

In cancer metastasis, tumor cells must acquire invasive and migratory phenotypes before they can metastasize. Khurana A, *et al.,* reported that Sulf-1 depletion by hypoxia inducible factor1 in hypoxic condition in breast cancer MCF10DCIS cells resulted in increased migration and invasion abilities, whilst on the other hand, its expression inhibited the bFGF signaling and led to reduction in migration and invasion of the breast cancer cells [39]. Similarly, our findings, both in vivo and in vitro, showed that Sulf-1 up-regulated cells had significantly reduced migration, invasion and LNM rate compared to the controls (Figure 4).

In addition to this, immunohistochemistry staining showed that Sulf-1 expression in Sulf-1-Hca-F group was much higher than in the two controls and at the same time the expression of Msln decreased in Sulf-1-Hca-F group which was consistent with the vitro findings. Furthermore, reduction in tumor growth and LNM were observed in Sulf-1-Hca-F group compared to the two control groups, which means that Sulf-1 played a role in the inhibition of tumor growth as well as reduction in lymphatic metastasis.

## CONCLUSION

The relationship between Sulf-1 and Msln has never been addressed by any investigation before, so our study for the first time showed that there is a functional relationship between the two genes in hepatocellular carcinoma. The tumor suppressor function of Sulf-1 and the tumor promoter role of Msln have been confirmed in this study, both in vitro and in vivo. In addition, we have demonstrated that overexpression of Sulf-1 reduces the expression of Msln, and this resulted in a decrease in HCC cell proliferation, migration, invasion, and lymphatic metastasis. Therefore, in our strive to get a better understanding and a cure for HCC, more research is required to further explore the relationship between sulf-1 and Msln. This exploitation could be a key in unlocking a novel strategy towards finding a cure for HCC which is one of the diseases with a high case fatality rate.

## MATERIALS AND METHODS

### Cell lines and stable plasmid transfection

Mouse hepatocellular carcinoma Hca-F (established by department of pathology, Dalian Medical University, Dalian, China) were cultured in 90% RPMI 1640 supplemented with penicillin/streptomycin and 10% fetal bovine serum (Gibco, USA), and cultured in a humidified incubator at 37°C with 5% CO_2._

Hca-F cells were divided into 3 groups; (1) Sulf-1 expression plasmid in Hca-F cells (Sulf-1-Hca-F) (2) nonspecific sequence control plasmid in Hca-F cells (Nc-Hca-F) (3) un-manipulated Hca-F cell line (Hca-F). A day prior to transfection, 5× 10^5^ cells per well was plated into a 6-well plate in a medium with 10% FBS and placed in an incubator with a temperature of 37°C with 5% CO_2_. Then the cells were stably transfected with Sulf-1overexpression plasmid (Sulf-1-Hca-F) and nonspecific sequence control plasmid (Nc-Hca-F). The non-transfected group was labelled as un-manipulated Hca-F cells (Hca-F). The transfection was done by using lipofectamine 2000 Reagent (Invitrogen, USA) according to the manufacturer's protocol. Prior to the transfection, we empirically determined G418 selection concentration to be 400 μg/ml of effective drug concentration, and after 72hrs post-transfection the cells were cultured in a medium containing this pre-determined G418 concentration until the entire cells in the non-transfected wells died off. We continued to culture the cells under the selection pressure of the G418 until they became completely resistant to the drug. The stably transfected cells were then cultured and the level of Sulf-1 protein up-regulation was determined by Western Blot procedure.

### Western blot analysis in vitro and in vivo

Cells in vitro in the log phase of growth were harvested and washed twice with ice-cold PBS whilst the in vivo tissue samples were homogenize using tissue homogenizer. Total cell and tissue sample proteins were extracted and then quantified by the BCA method using Nanodrop spectrophotometer (Thermofisher Scientific USA). Equal amounts of proteins prepared into equal volumes were loaded onto a gel (SDS-PAGE) and separated by electrophoresis. Guided by a pre-stained protein molecular weight ladder, portions of the gel corresponding to the molecular weights of Sulfatase-1, Mesothelin and Glyceraldehyde 3-phospate dehydrogenase (Gapdh) proteins were sectioned out and transblotted onto a PVDF membrane (Invitrogen, USA). The membrane was blocked in 5% non-fat dried milk for one hour and then probed with monoclonal goat anti- Sulfatase 1 antibody (Abcam, China, 1-3μg/ml), anti- Mesothelin antibody (Abcam, China, 1μg/ml) and Gapdh (ZSGB-Bio, China, 1:7500) primary antibodies for 1hr. After washing the membrane six times, rabbit anti-goat secondary antibody was applied to Sulf-1, anti- rabbit secondary antibody applied to Msln and anti-mouse secondary antibody applied on Gapdh for 1hr and the bright bands were captured by Li-Cor Odyssey Infrared Imaging System (Version 3.0 software).

### qRT-PCR analysis in vitro and in vivo

In the in vitro experiment, total RNA was extracted from Sulf-1-Hca-F, Nc-Hca-F and Hca-F cell lines using Trizol (Invitrogen, USA) according to the instructions of the manufacturer. In the in vivo experiment, prior to mRNA extraction, all the equipment was autoclaved and washed with nuclease-free water, followed by rinsing with 100% ethanol and left to air dry. The samples were thawed and 1g of cancer tissue was measured from each of the groups. The samples were homogenized using tissue homogenizer and total RNA was extracted from 500mg of the homogenate using the Trizol method. Reverse transcription of purified RNA from both the in vivo and the in vitro samples were performed using oligonucleotide dT primer. qRT-PCR was carried out using SYBR green I dye and the quantification of gene transcripts was performed and normalized to Gapdh as the internal control. The sequences of primer pairs used in the present study are listed in Table 3. PCR was carried out under the following conditions: 45 cycles of denaturation for 30sec at 95°C, annealing for 30sec at 55°C, and extension for 30sec at 72°C, and the relative mRNA expression level was collected and measured by using ^Δ^Ct equation. The PCR was carried out with Mx 3005P qRT-PCR machine (Agilent Technologies, Germany).

**Table 3:** Nucleotide sequences of the primers used in qRT-PCR

Genes	Primers	
	Forward	Reverse
Sulf-1	GCCAAGCGCCATGATGAG	TTCCACGCTCTGGCTGACT
Msln	CACACTGAAAACTCTGCTCAAAGTC	TCACATAGATAGCTTAACGGGATGTC
Gapdh	AAGGGTTTGGGACAGACGA	CATGAACAGCGCAAGGATTA

### Cell proliferation analysis in vitro

The effects of Sulf-1 up-regulation on Hca-F cell proliferation were measured using Dojindo's CCK-8 cell proliferation kit (Dojindo Molecular Technologies, Japan). Briefly, triplicates of 3× 10^3^ cells/well of Hca-F, Sulf-1-Hca-F, and Nc-Hca-F cells were plated in 96 well plates and Cell proliferation was measured at 24hrs, 48hrs, 72hrs and 96hrs later. The Cell proliferation test was done by adding 10μl of CCK-8/well and the absorbance was measured 30min after adding the reagent at 450nm using Multiskan Go spectrometer (Thermofisher Scientific, USA).

### Flow cytometry analysis in vitro

The three groups of cells (Hca-F, Sulf-1-Hca-F and Nc-Hca-F cells) were synchronized at G_0_/G_1_ phase by growth in 100% confluence with reduced serum for three days [21]. The cells were then passaged and cultured for 24hrs after which they were harvested in the log phase of growth, washed twice with ice-cold PBS and fixed in 75% cold ethanol overnight at 4°C. The following day the cells were washed twice with ice-cold PBS after discarding the ethanol, following which 50μg/ml of RNase (Sigma, USA) was added for 30 min and then stained with 20μg/ml of propidium Iodide (Sigma, USA) overnight in darkness. The cells were analyzed by flow cytometry (Beckman Coulter, USA) and the data were analyzed by Multicycle software (Phoenix Flow Systems, San Diego, USA) to get the cell cycle distributions.

### Migration assay in vitro

Transwell cell culture plates were used to determine the extent to which Sulf-1 up-regulation has inhibited the cells' migration potential. The upper chambers of the inserts were seeded with 2× 10^4^ cells in 200 μl serum-free 1640 whilst the lower chamber was filled with 750μl of 1640 containing 20% FBS as a chemoattractant, except in control wells which contained serum-free 1640 in both upper and lower chambers. After 16hrs of incubation in humidified incubator with 5% CO_2_ the non-migrated cells in the upper chamber were swabbed off and the plates were fixed, stained, and then observed under an inverted fluorescent microscope. Cells in five microscopic field views of representative areas in each of the groups were counted and averaged.

### Cell invasion assay in vitro

The inner chambers of the transwell plates were coated with ECM gel (Sigma, USA) and incubated at 37°C for 1hr to produce an artificial basement membrane. The rest of the procedure was as described in migration assay above. Both the migration assay and the invasion assay were performed concurrently, and the former, aside being an assay on its own, was additionally used as control for the later.

### Animals and implanted cell lines in vivo

The animals were provided by the Animal Facility of Dalian Medical University. All experimental procedures were approved by the Animal Ethics Committee of the Dalian Medical University, China. A total of 21 male inbred 615 mice (aged 6-8 weeks, weighing 18-22g) were randomly divided into three groups. The left footpad of each mouse was inoculated with 0.1ml cell suspension (approximately 2×10^6^cells).

### Tumor growth and lymphatic metastasis assay in vivo

Tumor growth was monitored and measured on days 7, 14, 21 and 28 and the values were used to calculate the tumor volume according to the formula [(length (mm) × width (mm)^2^)]/2. Four weeks later, all the mice were humanely sacrificed to dissect and weight the tumor and also to dissect the reginal lymph nodes (inguinal and axillary) for lymph node metastatic rate analysis. Some of the tumor tissues were fixed in 10% formalin for subsequent histological analysis whilst other tumor tissues were refrigerated for immunohistochemistry, western blot and qRT-PCR analysis.

### Immunohistochemistry (IHC) and hematoxylin and eosin staining analysis

The resected tumors tissues were cut into 5-μm sections and fixed in paraformaldehyde. IHC stain was employed to detect the expression of Sulfatase-1 and Mesothelin. The tissues were fixed in 4% buffered formalin for 15 min at 4°C and rinsed in TBS. paraffin sections were dewaxed in xylene and rehydrated in a series of ethanol solutions after which antigen retrieval was done with Tris buffer at pH 9.0 in a microwave. Endogenous peroxidase activity was blocked by 20 min pre-incubation with 3% H_2_O_2_ and then incubated with the blocking solution (Horse serum) for 30 min at room temperature. The incubation with primary antibodies (goat monoclonal anti- Sulfatase 1 antibody, Abcam, China, 1-3μg/ml, and anti- Mesothelin antibody, Abcam, China, 1μg/ml) was carried out overnight at 4°C. it was then Incubated with the secondary antibody for 1hr and color was developed with diaminobenzidine (DAB) (Zhongshan Biotechnology, China). The positive reaction manifested as a brown stain. The section was counterstained in Mayor's haematoxylin. Dehydration process started from 80%, 95% and 100% of ethanol each for 2 min and then washed with xylene 2times for 2 min each. Mounting and cover slipping were done and we proceeded to slide reading. Other slides were stained by routine Haematoxylin and Eosin staining method and the rate of lymph node metastasis was determined.

### Statistical analysis

Each assay was performed three times. SPSS 17 software was used for all statistical analysis. One-way Anova was used to determine the significant differences among the groups at *P* < 0.05. The data were expressed as the mean ± standard deviation (S.D).
